# Computational methods for processing and interpreting mass spectrometry-based metabolomics

**DOI:** 10.1042/EBC20230019

**Published:** 2024-04-30

**Authors:** Leonardo Perez de Souza, Alisdair R. Fernie

**Affiliations:** 1 Max Planck Institute of Molecular Plant Physiology, Am Mühlenberg 1, 14476 Potsdam-Golm, Germany; 2 Center for Plant Systems Biology and Biotechnology, 4000 Plovdiv, Bulgaria

**Keywords:** bioinformatics, machine learning, mass spectrometry, metabolomics

## Abstract

Metabolomics has emerged as an indispensable tool for exploring complex biological questions, providing the ability to investigate a substantial portion of the metabolome. However, the vast complexity and structural diversity intrinsic to metabolites imposes a great challenge for data analysis and interpretation. Liquid chromatography mass spectrometry (LC-MS) stands out as a versatile technique offering extensive metabolite coverage. In this mini-review, we address some of the hurdles posed by the complex nature of LC-MS data, providing a brief overview of computational tools designed to help tackling these challenges. Our focus centers on two major steps that are essential to most metabolomics investigations: the translation of raw data into quantifiable features, and the extraction of structural insights from mass spectra to facilitate metabolite identification. By exploring current computational solutions, we aim at providing a critical overview of the capabilities and constraints of mass spectrometry-based metabolomics, while introduce some of the most recent trends in data processing and analysis within the field.

## Introduction

Omics technologies have revolutionized biological sciences by providing comprehensive insights into the intricate molecular workings of living systems. Among these, metabolomics stands out - being the closest link to phenotype and thereby representing a powerful tool for understanding cellular dynamics.

The vast chemical diversity of metabolites, with in excess of a million distinct chemical structures thought to be present across extant organisms [1,2], requires advanced analytical methods, and both the identification of unknown metabolites and the comprehensive coverage of the metabolome remains a great challenge. The diversity of structures exhibiting widely diverse physicochemical properties, complicates the achievement of comprehensive coverage using a single analytical technique. In contrast with nucleic acids and proteins that present regular structures built from the linear arrangement of a finite set of building blocks, the lack of structural regularity among metabolites results in a vastly larger search space of plausible structures. In fact, even considering only simple organic molecules such as alkanes, with a general molecular formula of C_*n*_H_2*n* + 2_, a 20-carbon molecular formula can be assigned to over 3.3 million different stereoisomers [3].

Few analytical tools have the necessary features to tackle this challenging task. Among them, the integration of ultra-high-performance liquid chromatography (UHPLC) and high-resolution mass spectrometry provides the best coverage [4]. It provides high sensitivity to detect lowly abundant compounds, high dynamic range to cover a broad range of concentrations, high resolving power to allow quantification of multiple compounds in a complex matrix, and enough structural information to retrieve putative identities of measured metabolites. Additionally, LC-MS is the most flexible of all available technical platforms and can be adjusted for the detection of a wide variety of different compounds. Still, it is important to stress that no sole technique can cover the whole metabolome, and that much of what we can currently measure remains unidentified. Next, we discuss computational approaches that have been fundamental in assisting the interpretation of the complex datasets generated by LC-MS metabolomics experiments. They focus largely on two essential topics for exploratory metabolomics experiments. First, the unbiased detection of as many metabolites as possible. Second, the annotation of the large proportion of the metabolome that remains unidentified, colloquially termed the ‘dark’ metabolome.

## LC-MS data overview

**Figure 1:**
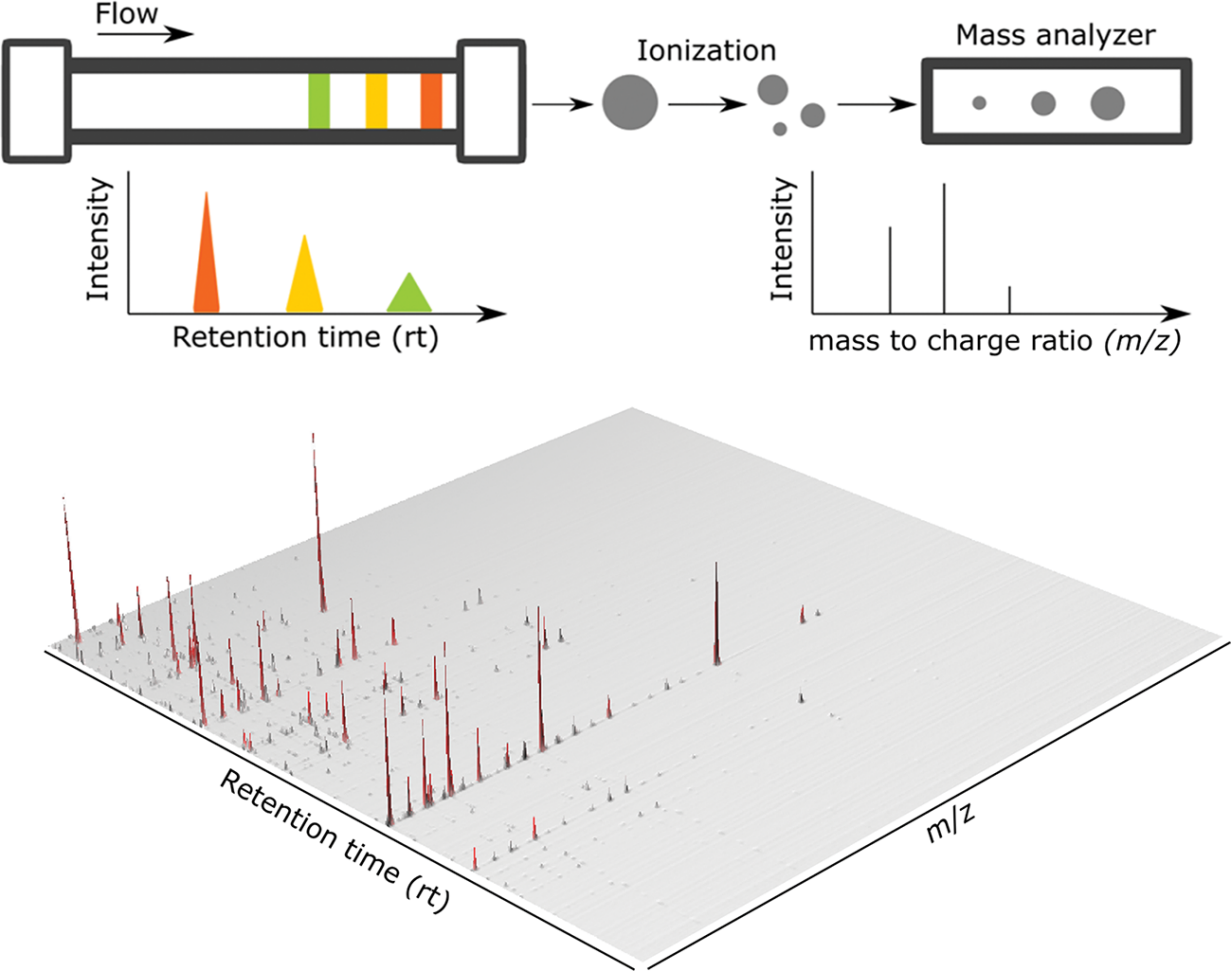
Exemplary overview of LC-MS mechanism and resulting mass chromatogram In the chromatographic separation (top left) a mixture of compounds, represented by the different colors, elutes through the column. The differential interactions of the compounds with the mobile and stationary phases within the column separates them based on their retention time as represented by the differentially colored peaks in the chromatogram. All the efflux of the chromatographic system is ionized and ions are separated based on their mass to charge ratio (*m/z*) within the mass spectrometer (top right). The resulting multidimensional mass chromatogram is represented in the lower panel.

In a typical LC-MS run, compounds within a mixture differentially interact with the phase within a chromatographic column leading to their separation at distinct retention times (rt - glossary) (Figure 1). Analytes eluting off the column, are directly ionized via electrospray ionization (ESI). This process usually yields a mixture of the protonated molecular ion (depronated in negative ionization mode), as well as common adducts (glossary) formed with components of the mobile phase and environment (e.g. formic acid, acetic acid, Na^+^, and K^+^), and *in source* fragments (glossary). Subsequently, these ions make their way into the evacuated analyzer where they are separated based on their mass to charge ratio (*m/z*), prior to being registered by a detector. An additional step of fragmentation, termed tandem MS, is usually included inside the mass spectrometer providing second order (MS^2^) spectra. MS^2^ spectra provide the fragmentation pattern of the selected precursor ion, which is of great interest for structural elucidation. The resulting mass chromatograms emerge as highly complex multi-dimensional datasets, necessitating sophisticated computational strategies to fully leverage the biological insights attainable from them.

## Data processing tools

Traditionally, manual data processing involves extensive and time-consuming assessment of the mass chromatograms. *In source* fragments and adducts must be identified and grouped for the selection and quantification of a single representative ion per analyte. This is the first computationally intensive process, and has been largely automated with the help of signal processing tools, and implementation of heuristic rules previously used by the analyst to classify detected signals. Several free data processing tools are available whose primary function is to automatically detect mass features generating well defined chromatographic peaks, and compare peak intensities across multiple samples, as a proxy for metabolite concentration. Among some of the most popular are XCMS [5], mzMine [6,7], OpenMS [8], and MS-DIAL [9]. For a more extensive list of the myriad of tools for this purpose we refer the reader to the more comprehensive reviews of Perez de Souza et al. [10] and Misra [11].

The automation of this peak processing step undoubtedly improves the unbiased analysis of the data but imposes some new challenges. *In source* fragments and adducts generate multiple redundant signals in the mass chromatograms. This is a major source of multicollinearity (glossary) in metabolomics datasets that should be removed to improve data analysis. There are several tools, based on correlation over narrow rt windows and automatic detection of relevant *m/z* differences, that can assist this annotation and often integrate directly with the aforementioned tools. These include CAMERA [12] and MS-DIAL [9]. However, a considerable effort in manual data curation is still required to access the results.

## Processing optimization and curation

Another consequence of unbiased signal processing is the large proportion of poorly integrated signals. Automated processing of such complex datasets usually involves tradeoff between processing method sensitivity and the quality of the integrated data. Therefore, selecting the multiple parameters available for all the aforementioned tools is far from trivial. A few tools have tried to implement systematic methods for parameter optimization [13,14], and more recently, a dedicated processing tool (SLAW) was developed to provide self-optimizing processing workflows [15].

Considering how variable and sensitive data processing is, it is generally good practice to evaluate a workflow based on some previous knowledge of the sample, or some internal controls. A very interesting tool that facilitates this performance assessment is mzRAPP [16]. mzRAPP generates a benchmark subset from the analyzed dataset and uses it to return well-defined processing performance metrics.

Despite the availability of all these resources, careful curation of the processed data is strongly recommended. Such curation aims at classifying automatically processed data into poorly or well-integrated/defined peaks, removing the former from downstream analysis. Unsurprisingly, as any classification task, this can profit from modern machine learning tools. A few deep learning algorithms have been released that allow for a relatively quick dataset specific training based on user input of good and poorly integrated peaks [17–19]. These algorithms allow for the removal of such features with great success.

## Second order spectra

Fragmentation patterns, together with accurate mass, provide the most important information regarding compound identification in mass spectrometry. It is important to highlight that *in source* fragmentation is still considered mostly a detrimental factor, since it has low reproducibility and increases dataset complexity. Still, it can provide some structural fragmentation, and there are attempts to explore it [20]. The use of tandem MS experiments is a preferred strategy though, providing more reproducible and interpretable data.

**Figure 2:**
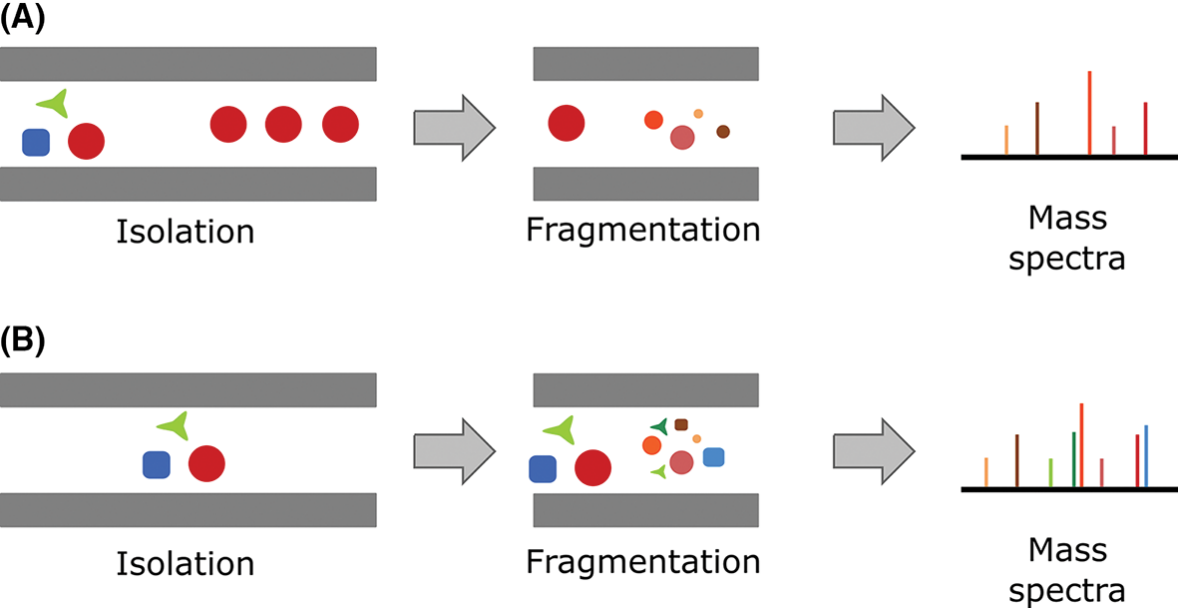
Diagram representing tandem MS experiments Diagram representing tandem MS experiments in (**A**) DDA, and (**B**) DIA modes. The different geometric forms in the isolation chart represent a mixture of different ion populations and how they are transferred through the device on the different data acquisition modes. In (A) only the ion population represented by the red circle make their way through and is fragmented. All smaller fragments represented in the fragmentation chart are product ions of the same isolated precursor. In (B), all the different ion populations make their way through the isolation device and are fragmented. The smaller fragments represent product ions of one of the three different precursor ion populations within the fragmentation chart. The resulting mass spectra registered are visually similar, however, for (B) the precursor/product ion relationships are unknown.

A traditional tandem MS experiment is performed inside the mass spectra, with target ions being isolated and fragmented. This traditional setup is often referred to as data dependent acquisition (DDA), given the relationship between the fragments (also known as daughter ions) and the original ion (also known as parental ion) is well stablished (Figure 2A). The isolation of each individual ion from the MS1 spectra demands scanning time from the detector. Therefore, the comprehensive coverage of all MS1 signals is normally impossible. Most metabolomics-oriented methods use the strategies of either automatically selecting TopN most intense ions on each MS1 scan event for immediate MS2 fragmentation in successive scans, or they perform an in-depth fragmentation study following repeated injections of representative samples. The former sacrifices coverage, while the latter sacrifices experiment throughput, since it demands extensive manual tuning of successive runs and posterior integration of data across different raw files to characterize a single sample.

Data independent analysis (DIA), in contrast, provides comprehensive fragmentation of all MS^1^ ions, by using very large or no isolation windows, and fragmenting a mixture of multiple MS^1^ ions (Figure 2B). The result is a highly multiplexed MS^2^ spectra, with no clear precursor-product ion relationships, demanding considerably more computational effort to re-construct these relationships, and resulting in overall lower quality second order spectra. Most of the popular data processing tools, are currently developing and integrating alternatives to process DIA data, highlighting MS-DIAL that was developed with a particular focus toward that [21].

In an ideal scenario, in-depth analysis to obtain DDA data for most of the ions is the preferred approach. However, it is important to recognize the limitations imposed by the reality of most metabolomics facilities and experiments. Equipment time availability and the need for high throughput in order to run large association studies, for example, can impose significant limitations. In such situations, approaches such as TopN and DIA, provide the flexibility necessary to acquire valuable structural information while minimizing extensive fragmentation studies.

## Metabolite annotation – spectral database search

After completing the initial data processing, the path to follow depends significantly on the goal of the experiment. Most general exploratory experiments, seek to comprehensively characterize the metabolic composition of the samples. In the following sections, we will focus into some essential concepts and tools that facilitate such untargeted and unbiased global annotation.

The most widely adopted approach for metabolite annotation relies on MS^2^ spectral database searches. At the core of such methods lies the concept of quantifying the similarity between an experimental spectrum and that of an authentic standard stored within a spectral database [22]. The most commonly used score is the dot product, or cosine similarity [23]. This similarity measure computes the cosine of the angle between the two vectors representing each spectrum, ranging from 0 to 1, when both spectra are identical. It serves as an efficient metric to compare spectra with relatively high degree of purity. An alternative, often useful for contaminated spectra commonly found in metabolomics experiments, is the use of the reverse dot product, which omits from the calculation all peaks that do not match in the query spectra [22,23].

Although spectral database search appears straightforward, it faces significant limitations from two key factors. First, isomers frequently produce remarkably similar, often indistinguishable spectra. Second, the search is constrained by the relatively limited number of compounds available within spectral databases. The former challenge can be considerably amended by incorporating retention time information for dereplication of identical spectra, despite the great challenge that is to map retention times across different platforms. Quantitative structure-retention relationship (QSRR) models constitute a promising strategy to predict retention times and elution order of different compounds across systems [24]. Incorporating QSRR-based predictions as an additional scoring has a great potential aiding the selection between equally likely candidates based solely on spectrum similarity.

## Metabolite annotation – network analysis

Perfect spectral database matches are not only rare due to the intrinsic variations of data generation, but also due to the huge diversity of the metabolome, for which only a minute proportion is represented with spectroscopic data in standard databases. Unsurprisingly, one area of metabolomics research that has observed growing interest in the recent years, is without question that of new compound elucidation. As highlighted by many authors, the vast majority of the detectable metabolome remains uncharacterized. This represents a huge untapped resource as many large-scale experiments rely on metabolite annotations to drive biological and mechanistic conclusions, often leaving aside strong associations with unknown compounds. Therefore, several alternative metabolite annotation approaches try to expand the reach of mass spectrometry-based annotation beyond previously characterized molecules.

Molecular networking is one such approach that became particularly popular since its introduction [25] and the later implementation of the GNPS platform for data analysis and community sharing of the annotated results [26]. The core idea behind molecular networking is again based on similarity scores, but here they are used to stablish connections between unknown experimental spectra that share scores above a certain threshold. The interpretation of these networks under the assumption that similar structures generate similar spectra allows for the propagation of a few characterized metabolites throughout the dataset. Regarding the similarity score, molecular networking traditionally implements a modified dot product that better captures similarities between spectra from compounds that undergo small functional changes [25,27]. This is achieved by accounting not only for perfect matches, but also for pairs of fragments that differ in the same *m*/*z* as the precursor ions.

Since the popularization of molecular networking several sophisticated network-based approaches for metabolite annotation have been developed extending these ideas. Many of these, such as the use of precursor mass differences to infer biochemical transformations [28], and *in silico* fragmentation are highly complementary [29], and some have been integrated within the GNPS environment through MolNetEnhancer [30]. Others remain as stand-alone tools, but integrating multiple network layers. NetID [31] provides a global network optimization approach using linear programming that incorporates information regarding MS2 spectra similarity, precursor mass shift, and retention time shifts. This strategy allows for the identification of putatively related compounds whilst also providing information regarding the biochemical relationships and identifying putative *in source* fragments and adducts. MetDNA [32,33] is in its second iteration, and similarly combines spectra similarity, knowledge based metabolic reactions, and correlations to annotate both putative compounds and potential adducts and fragments.

Establishing these connections between metabolites is also very useful from a data analysis and biological interpretation perspective. Many pathway enrichment tools such as metabolite set enrichment analysis, rely on pathway databases and therefore are mostly constrained to central metabolism. However, most of the metabolic diversity that characterizes the metabolome is not captured by such pathway databases, often being species specific or at least of limited distribution. Similarity and correlation networks can be used to implement enrichment analysis with little to no information regarding metabolite identities [34].

## Metabolite annotation – *in silico* structure prediction

*In silico* structural predictions for *de novo* structural elucidation has long been a goal of metabolomics to overcome the limited metabolome coverage of spectral library databases. Recent developments in computational power, access to large high-quality datasets and machine learning, have paved the way to exciting developments in the past few years. In this scenario two contrasting conceptual frameworks have been explored, the first is translating molecular structures into spectra, while the second revolves around precisely the reverse process of translating mass spectra into molecular structures that are not yet present in databases [35].

Chemical structure databases such as PubChem [36] and Coconut [37] provide a vastly larger coverage of known chemical structures in relation to spectral databases, with the downside of lacking spectral information for most of them. Initial approaches to leverage the much higher coverage of these structural databases involved the development of tools that attempt to model the processes happening in the mass spectrometer to predict fragmentation solely from a chemical structure [38]. *In silico* fragmentation tools rely on diverse approaches including the combinatorial fragmentation of molecular bonds, and application of heuristic rules of fragmentation. Popular tools such as MAGMa+, MetFrag and MS-Finder [39–41] rely on different implementations of either one or a combination of both these approaches. Molecular fragmentation based on quantum chemistry calculations has also been explored as a possible solution for *in silico* fragmentation, and are available with tools such as QCMS^2^ [42] and ChemFrag [43]. However, these methods demand considerable computational power which makes them less viable for the scale demanded by metabolomics studies. Machine learning models have also been used for *in silico* fragmentation. Some of the pioneering work on this field has culminated into the CFM-ID model [44]. CFM-ID predicts break tendencies of possible fragments and translates them into probabilities to grasp the competitive dynamics among potential breaks within the same molecule [38]. More recently, GRAFF-MS model has shown promising improvements with lower prediction error and considerably faster runtime using graph neural networks [45].

Alternatively, the prediction of molecular fingerprints from mass spectra has also been explored [46,47]. A molecular fingerprint is a long vector that encodes structural features of a molecule and it is a popular tool in chemoinformatic facilitating calculations involving molecular structures [48]. One of the best performing tools for metabolite annotation available in the past years, CSI:FingerID [47], integrated in the Sirius platform [49], uses predicted molecular formulas and fragmentation trees to generate a fingerprint and match this against structural databases. The main limitation of these approaches is that they are still restricted to the now much larger structural databases. Attempts to amend this dependency have shown promising results such as the integration of *in silico* structure database generation represented by the COSMIC workflow [50], however it still faces computational limitations once the chemical space explored becomes too large.

Recently, attempts to generate models capable of translating mass spectra into structures, dispensing the need of any database, seem to have finally taken off with the release of MSNovelist, Spec2Mol, MassGenie and MS2Mol [51–54] in the past 3 years. These methods have profited significantly from the development of deep learning methods to molecule generation such as the pioneering work by Gómez-Bombarelli et al. [55]. Perhaps it comes with no surprise that the machine learning models incorporated in these tools are also widely employed in natural language processing. Mass spectra analysts have long interpreted the fragmentation patterns in a similar way to a language. In fact, text mining has inspired spectra interpretation tools in the past. MS2LDA [56] for instance facilitates the extraction of fragmentation patterns linked to specific functional groups by leveraging latent Dirichlet allocation, originally designed to break down text documents into topics through the analysis of co-occurring words. Latest break throughs in natural language processing are likely to seamlessly translate into the field of mass spectral interpretation, significantly enhancing the efficiency of *de novo* structural prediction in the years to come.

## Outlook

Mass spectrometry is a continuously evolving technique that has seen remarkable advancements in recent years. These advancements have resulted in significant enhancements in mass resolution, sensitivity, and detector speeds. Consequently, mass spectrometry now enables more informative and comprehensive data collection than ever before. Concomitantly, improved computational power, access to data and developments in machine learning are bringing unprecedented advancements in some of the most challenging aspects of metabolomics data analysis.

Metabolomics has become essential to a variety of different fields and it is adopted by researcher with widely different background. User friendliness has been a determining factor towards the popularization of these computational tools through intuitive graphical user interfaces, cross platform integration, self-optimizing pipelines and quality assessment tools. Data annotation platforms such as Sirius and GNPS, and processing tools like XCMS, mzMine, OpenMS, and MS-DIAL are prime examples of how user experience is a fundamental aspect of bringing these good ideas into everyday practice.

Metabolomics experiments are typically conducted to address highly targeted research inquiries, resulting in data that often exceeds the immediate scope of the study. Consequently, a substantial portion of the generated data remains largely unexplored. The emerging trend within the scientific community to openly share extensive raw datasets, along with the development of repository-scale data analysis pipelines, holds great promise and excitement for the future. This forward-looking approach is expected to provide novel insights into the metabolome and its intricate interactions in the years ahead.

## Glossary

Adduct: An ion formed by interaction of two species within the ion source, often an analyte with components of the chromatographic solvent system, forming a single ion containing both constituents.

*In source* fragmentation: analyte fragmentation in the ionization source. It usually occurs as a byproduct of electrospray ionization.

Multicollinearity: Feature of a dataset where many variables are highly correlated. It usually results in a detrimental effect on statistical inferences.

Retention time (rt): The time a compound takes from the beginning of the run until it reaches the detector.

## Summary

- Recent advances in computational metabolomics allow for the processing extensive and intricate datasets, establishment of complex relationships and application of heuristic rules to effectively aggregate redundant signals.
- Machine learning provides efficient and significantly faster alternatives for data quality curation, eliminating poorly integrated signals from data processing.
- The advancement of spectral similarity scores and innovative applications across samples is facilitating the exploration of increasingly intricate associations and the propagation the scarce knowledge about annotated metabolites through similarity networks.
- *In silico* structure prediction is improving at a fast pace. The representation of molecular structures as fingerprints allow for sophisticated calculations establishing relationships between spectra and structure in both directions.
- New machine learning models inspired by language processing are promising tools to generate putative structures directly from mass spectra.

## Open Access

Open access for this article was enabled by the participation of Max Planck Digital Library in an all-inclusive *Read & Publish* agreement with Portland Press and the Biochemical Society.

## Abbreviations

DDA: data dependent acquisition
DIA: data independent analysis
ESI: electrospray ionization
LC-MS: liquid chromatography mass spectrometry
QSRR: quantitative structure-retention relationship
UHPLC: ultra-high-performance liquid chromatography
